# Obituary

**Published:** 1883-01

**Authors:** 


### Obituary.

Dr. Edmund Grindal Rawson, an old and highly respected physician, died at the age of 78 years. Dr. Rawson was born in Broad Albion, N. Y., on Nov. 30. 1803. He graduated from Union College in 1826. He then entered the College of Physicians and Surgeons, in this city, and was Assistant Physician in Bellevue Hospital in 1828 and 1829, afterward practicing medicine for two years in his native town. In 1832 he returned to this city, wherehe has been a successful medical practitioner ever since. Dr. Rawson was one of the incorporators of the New York College of Veterinary Surgeons, and was its President at the time of his death. He was also a member of the County Medical Society. In 1834 Dr. Rawson was elected to the Common Council of New York, where he served for several years. His death was caused by paralysis. He leaves a widow, but no children.

E. V. Ripley, V. S., of Portland, Maine, 58 years of age, died at Colorado Springs, where he had gone in the hope of restoring his failing health.

